# Self-sealing hyaluronic acid-coated 30-gauge intravitreal injection needles for preventing vitreous and drug reflux through needle passage

**DOI:** 10.1038/s41598-021-96561-8

**Published:** 2021-08-20

**Authors:** Youngsub Eom, Soomi Kim, Jungah Huh, Mi Young Koh, Jin Young Hwang, Boram Kang, Xiangzhe Li, Moon Sue Lee, Haeshin Lee, Hyo Myung Kim, Jong Suk Song

**Affiliations:** 1 Department of Ophthalmology, Korea University College of Medicine, 148, Gurodong-ro, Guro-gu, Seoul, 08308 Republic of Korea; 2 Department of Ophthalmology, Korea University Ansan Hospital, Gyeonggi-do, Republic of Korea; 3 R&D Center, InnoTherapy Inc, Seoul, Republic of Korea; 4 Department of Ophthalmology, Korea University Guro Hospital, 148, Gurodong-ro, Guro-gu, Seoul, 08308 Republic of Korea; 5 Blue Eye Center, Gyeonggi-do, Republic of Korea; 6 Department of Ophthalmology, The First Affiliated Hospital of Jinan University, Guangzhou, Guangdong China; 7 Department of Chemistry, Korea Advanced Institute of Science and Technology (KAIST), 291 University Rd., Daejeon, Republic of Korea

**Keywords:** Structural biology, Medical research, Outcomes research

## Abstract

Self-sealing hyaluronic acid (HA)-coated self-sealing 30-gauge needles exhibiting instant leakage prevention of intravitreal humor and injected drug were developed in this study. Ninety New Zealand rabbits were used in this study. We assessed dye regurgitation in intravitreal ICG dye injections using HA-coated needles (HA needle group) and conventional needles (control group). Vitreous humor levels of anti-vascular endothelial growth factor (VEGF) were compared between groups one, three, and seven days after intravitreal bevacizumab (0.016 mL) injections. Expression levels of inflammatory cytokines in the aqueous humor and vitreous humor, including prostaglandin E_2_ (PGE_2_), interferon-γ, tumor necrosis factor-α, interleukin (IL)-1β, IL-4, IL-6, IL-17, and IL-8, were compared between HA needle, control, and normal (in which intravitreal injection was not performed) groups following 12 intravitreal injections over a period of one week. In the HA needle group, HA remained at the injection site and blocked the hole after intravitreal injection. Dye regurgitation occurred significantly less frequently in the HA needle group (16.7%) than the control group (55.6%) after intravitreal ICG dye injection. Meanwhile, vitreous anti-VEGF levels were markedly higher in the HA needle group than the control group one and three days after intravitreal bevacizumab injections. After 12 intravitreal injections, expression levels of aqueous and vitreous IL-8 significantly increased in the control group compared to the HA needle and normal groups. Conversely, there were no significant differences in the expression of the other seven cytokines among the three groups. Intravitreal injections using HA-coated self-sealing 30-gauge needles can block the outflow of vitreous humor and drugs through the needle passage.

## Introduction

Intravitreal injection is widely used for drug delivery in the treatment of various diseases of the posterior segment of the eye. Antibiotics, anti-inflammatory drugs, and antibodies can thus be directly injected into the vitreous cavity for the treatment of ophthalmic diseases. Following intravitreal injections of steroids such as triamcinolone acetonide for the treatment of diabetic macular edema and uveitis, intravitreal injection of anti-vascular endothelial growth factor (VEGF) has become the most commonly performed intraocular procedure worldwide for treatment of age-related macular degeneration and other diseases^1,2^. As a result, intravitreal injection procedures are rapidly growing in frequency^3–7^.

Drugs injected into the vitreous cavity decrease in concentration according to their half-lives, which results in decreased efficacy over time^8,9^. As a result, intravitreal injections should be repeated at regular intervals or whenever recurrence is observed. Since repetitive intravitreal injections increase the risk of related complications, there is increased interest in these complications and their prevention^2^. Endophthalmitis can occur if bacteria enter the eye following needle passage for intravitreal injection^10^ and is one of the most devastating complications in ophthalmology, leading to blindness in severe cases^11–16^. Thus, there is a need to prevent infection following intravitreal injection^2,17^. Methods used to prevent endophthalmitis include using the beveled incision technique and performing the procedure in an aseptic environment, with disinfection using a povidone iodine solution before the procedure. The beveled incision technique could reduce vitreous and drug reflux compared to a straight incision, maximizing the drug delivery and decreasing the risk of infection^2,10^. On the other hand, regurgitation after intravitreal injection has the advantage of reducing intraocular pressure (IOP) after injection.

A previous study reported that, when vascular injections were performed with needles coated with a bio-inspired polymer, there was no bleeding after needle removal^18^. Therefore, intravitreal injection using needles coated with a bio-inspired polymer could have the advantage of preventing the outflow of vitreous humor and drugs. On the other hand, it can cause inflammation. Hyaluronic acid (HA) has been used as a biocompatible hydrogel material for anterior chamber delivery of cell sheet grafts^19^. More recently, HA-coated biomaterials have been shown to be safe after anterior chamber injection^20^. We therefore hypothesized that using needles coated with a substance such as HA, which can block the passage that forms after intravitreal injection, would prevent the outflow of vitreous humor and drugs, hindering introduction of pathogens through the needle passage site postinjection without causing inflammation. The purpose of this study was to verify the advantages and disadvantages of intravitreal injection using HA-coated needles in an animal study.

## Results

### Preparation of self-sealing intravitreal injection needles

**Figure 1:**
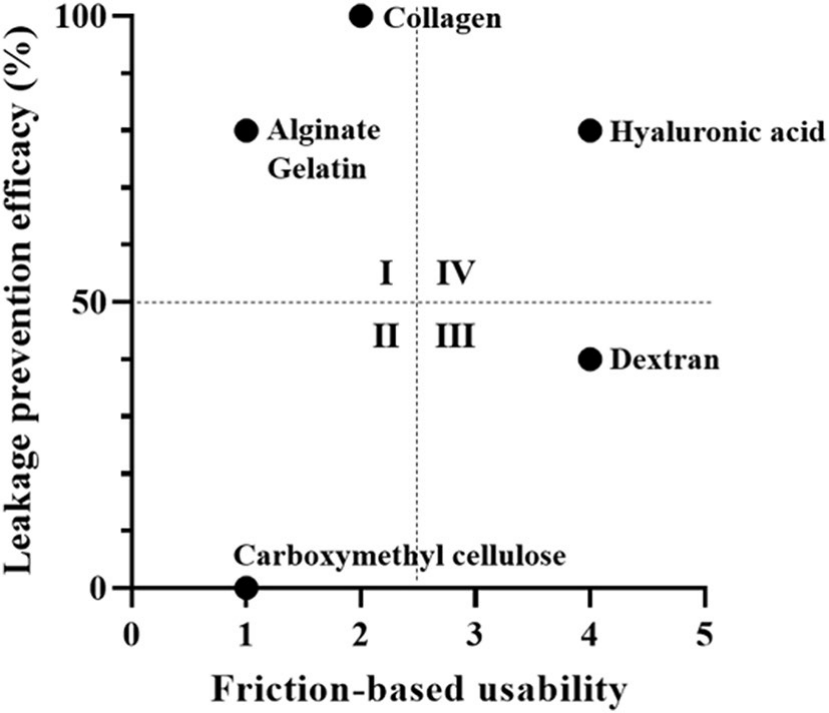
Optimization of coating polymers for self-sealing injection needles.

Collagen and HA showed satisfied results in the leakage prevention efficacy. However, the collagen coated needles exhibited high surface friction which was not a suitable for intravitreal injection uses (Fig. 1). The extreme molecular weights of 10-kDa and 1000-kDa HAs showed no prevention of leakage in ex vivo porcine eye leakage tests. In contrast, HAs with mid-molecular weights of 200 kDa and 700 kDa showed successful leakage prevention: 66.7% success rate for 200 kDa and 83.3% for 700 kDa. Based on these findings, the investigators selected HA 700 kDa for the experiments described in this study.

**Figure 2:**
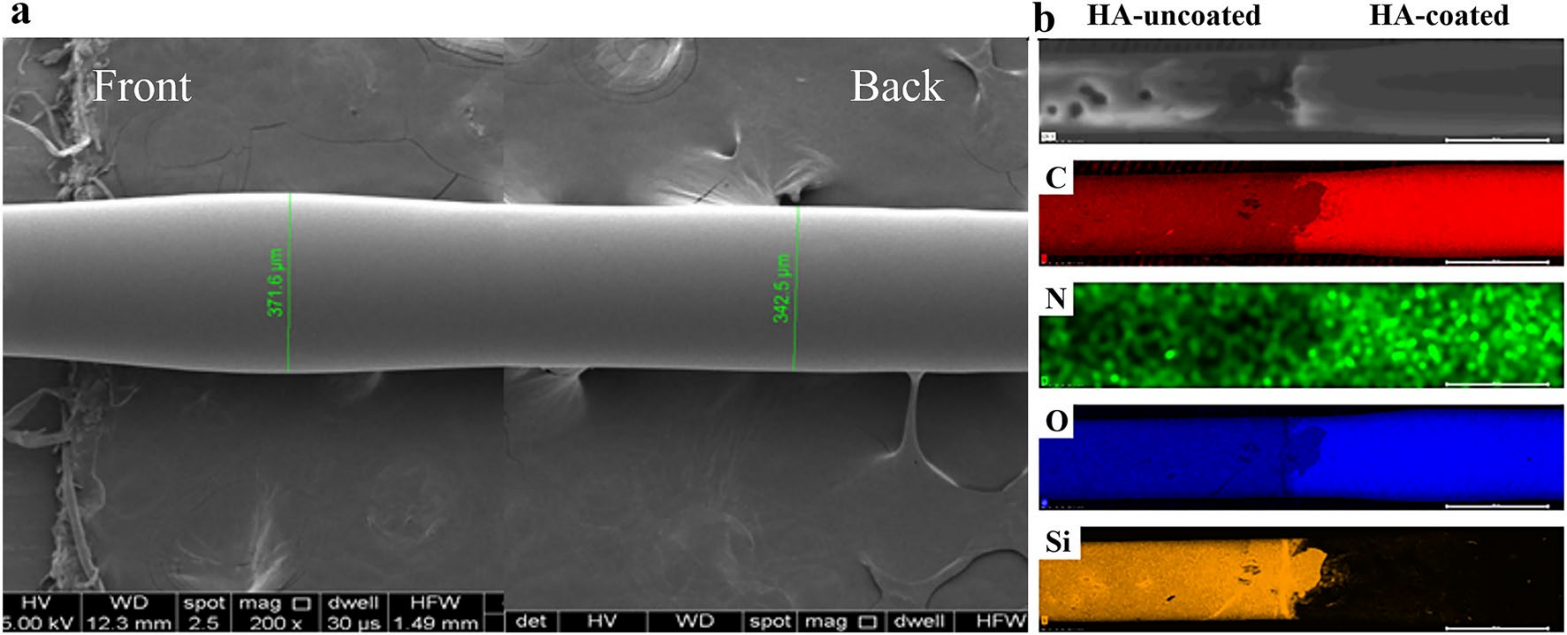
Hyaluronic acid-coated self-sealing intravitreal injection needles. (**a**) SEM image of the HA-coated self-sealing intravitreal injection needles. The thickness of the thickest part coated with hyaluronic acid was 371.6 µm and the thickness of the part not coated with hyaluronic acid was 342.5 µm. (**b**) EDS analysis of the HA-coated self-sealing intravitreal injection needles: Carbon (C, Red), Nitrogen (N, Green), Oxygen (O, Blue), and Silicon (Si, Yellow).

The thickness of the thickest part of the needle coated with HA was 371.6 µm and the thickness of the part not coated with HA was 342.5 µm (Fig. 2a). The area of HA coating, carbon (red), nitrogen (from N-acetylglucosamine, green), and oxygen (blue) signals were strong. The opposite side of uncoated areas, a silicon signal (yellow) was dominantly detected (Fig. 2b).

### Penetration test of ha-coated self-sealing intravitreal injection needles

**Figure 3:**
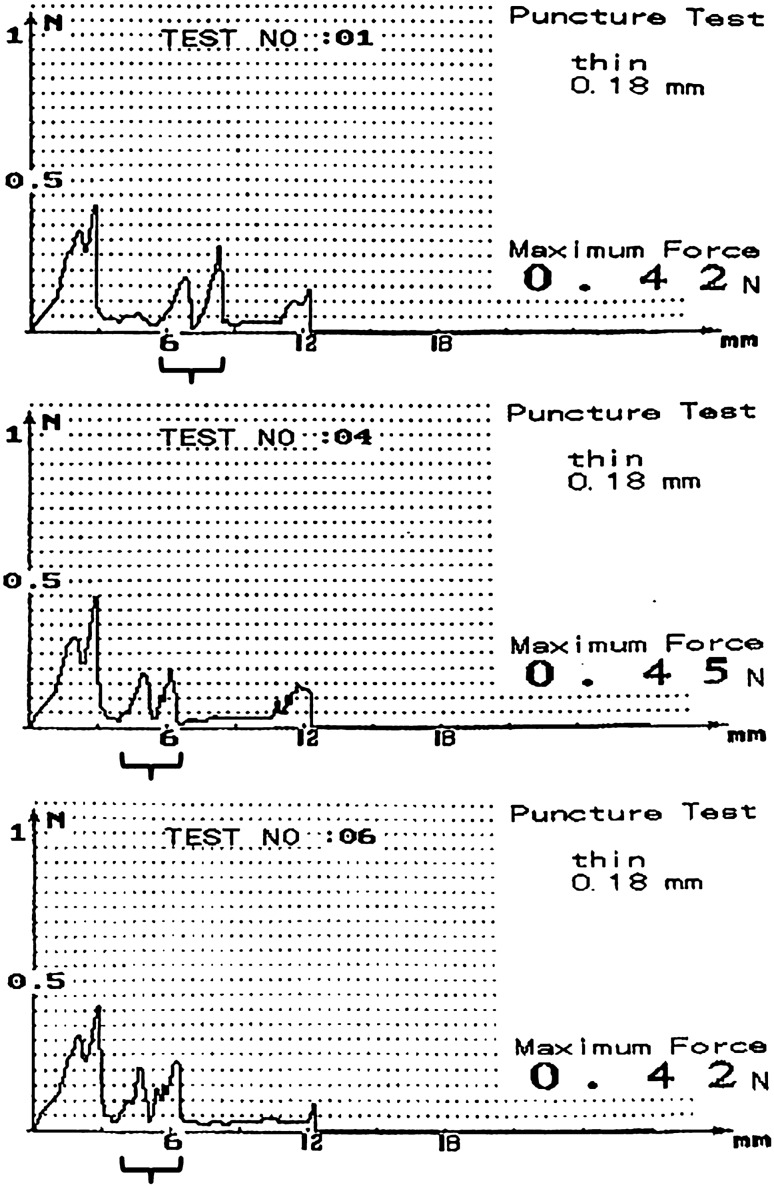
Results of penetration force tests of hyaluronic acid-coated self-sealing intravitreal injection needles. The strongest force produced when the HA-coated self-sealing injection needles penetrated artificial tissue was 0.42 N–0.45 N, while the penetration force at the position coated with the HA film was 0.20 N–0.30 N (braces).

Based on the results of needle penetration force tests, the strongest force produced when a HA-coated self-sealing injection needle (30 gauge × 1/2″; BD PrecisionGlide™ Needles, Singapore) with a 1-ml syringe (Jung Rim Medical Industrial Co. Ltd., Seoul, Republic of Korea) penetrated artificial tissue was 0.42 N–0.45 N, while the penetration force at the position coated with the HA film was weaker, measuring 0.20 N–0.30 N (Fig. 3).

### Immediate regurgitation of dye after intravitreal injection

**Figure 4:**
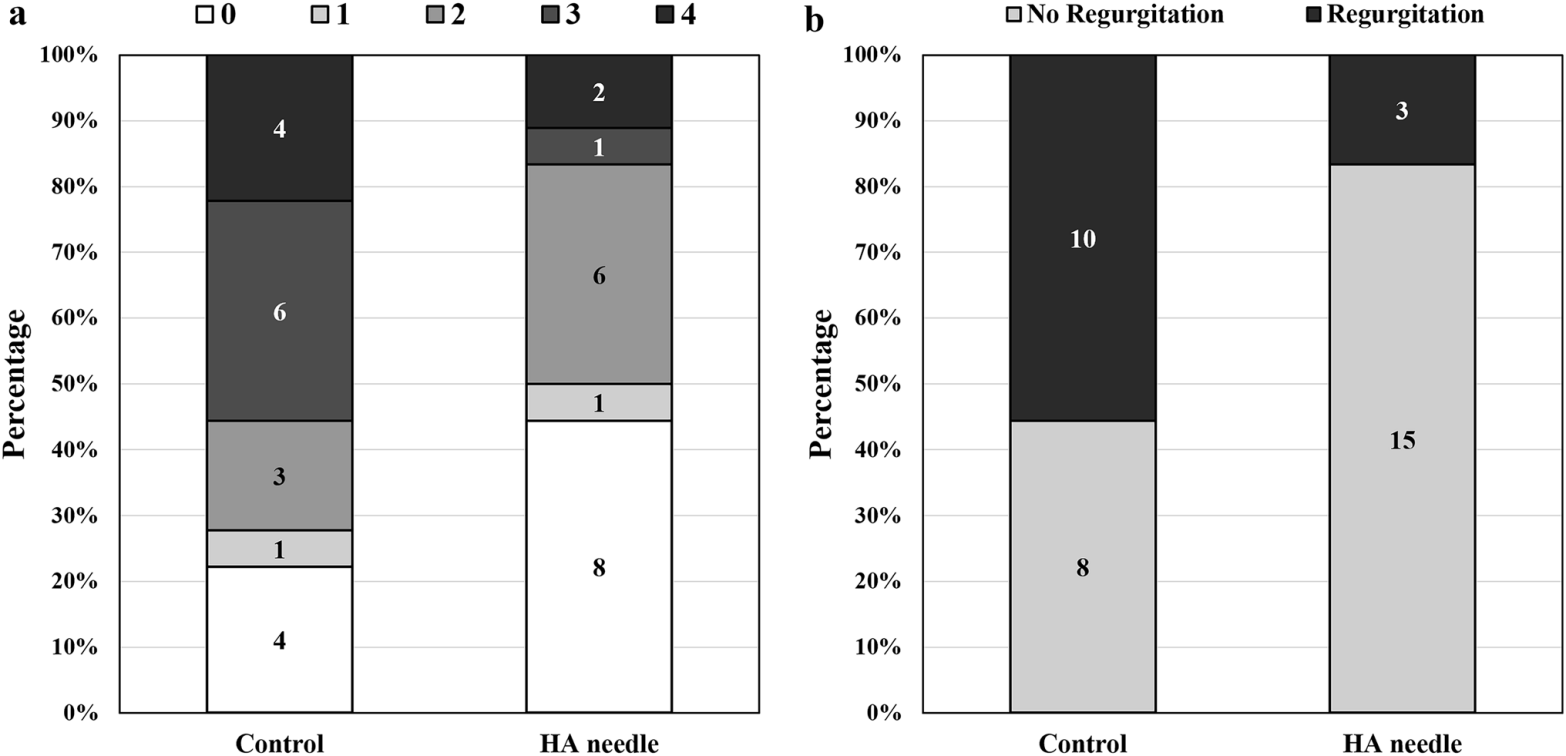
Evaluation of immediate regurgitation of injected dye after intravitreal injection. (**a**) Evaluation of staining status observed at the injection site conjunctiva according to stage (0, no stain; 1, dot stain; 2, spot stain; 3, chemosis; 4, leakage.). (**b**) Evaluation of dye regurgitation.

The staining status of the ICG dye was observed in the injection site conjunctiva immediately after intravitreal injection with dye using HA-coated or conventional needles. When the HA-coated needle was inserted and then removed, HA remained at the injection site and blocked the hole in most cases. For HA-coated needles, we observed eight cases of stage 0, one case of stage 1, six cases of stage 2, one case of stage 3, and two cases of stage 4. For conventional needles, we observed four cases of stage 0, one case of stage 1, three cases of stage 2, six cases of stage 3, and five cases of stage 4 (Fig. 4a). Three eyes (16.7%) in the HA needle group showed immediate dye regurgitation after intravitreal injection, which was significantly fewer than the 10 eyes (55.6%) observed to regurgitate dye in the normal group (*p* = 0.035; Fig. 4b).

### Aqueous and vitreous humor levels of anti-VEGF

**Figure 5:**
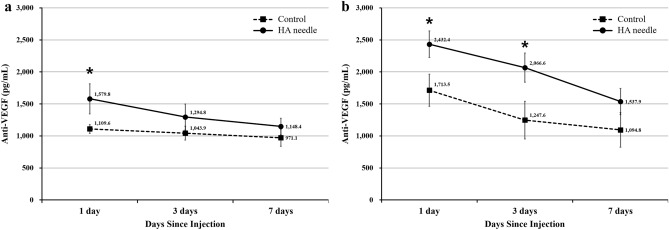
Comparison of changes in aqueous (**a**) and vitreous (**b**) anti-VEGF levels after intravitreal bevacizumab injection. The control group received 12 intravitreal injections over seven days using a conventional needle. The HA group received 12 intravitreal injections over seven days using a HA-coated needle.

The aqueous and vitreous anti-VEGF levels were 27.1 ± 8.7 pg/mL and 25.2 ± 16.2 pg/mL, respectively, in the normal group. Notably, aqueous anti-VEGF levels in the HA needle group after intraocular bevacizumab injection were 1579.8 ± 469.2 pg/mL, 1294.8 ± 404.2 pg/mL, and 1148.4 ± 257.5 pg/mL at one, three, and seven days, respectively. In the control group, these values were 1109.6 ± 137.5 pg/mL, 1043.9 ± 215.9 pg/mL, and 971.1 ± 267.1 pg/mL, respectively. The level of aqueous anti-VEGF was significantly higher in the HA needle group than the control group one day after injection (Fig. 5a and Table 1). Moreover, vitreous anti-VEGF levels in the HA needle group after injection were 2432.4 ± 415.2 pg/mL and 2066.6 ± 455.5 pg/mL at 1 and 3 days, respectively, markedly higher than the control group (1713.5 ± 500.5 pg/mL and 1247.6 ± 586.6 pg/mL, respectively). However, no significant difference was found at seven days between the two groups (Fig. 5b and Table 1).

**Table 1:** Comparison of aqueous and vitreous humor levels of anti-vascular endothelial growth factor (VEGF) between the control and hyaluronic acid (HA) needle groups.

	Control group	HA needle group	P value^a^
**Aqueous anti-VEGF level, pg/mL**			
1 day after injection	1,109.6 ± 137.5	1,579.8 ± 469.2	0.040
3 days after injection	1,043.9 ± 215.9	1,294.8 ± 404.2	0.210
7 days after injection	971.1 ± 267.1	1,148.4 ± 257.5	0.269
**Vitreous anti-VEGF level, pg/mL**			
1 day after injection	1,713.5 ± 500.5	2,432.4 ± 415.2	0.022
3 days after injection	1,247.6 ± 586.6	2,066.6 ± 455.5	0.022
7 days after injection	1,094.8 ± 538.8	1,537.9 ± 408.7	0.140

Data are expressed as the mean ± standard deviation (SD) of 6 rabbits.

The control group receiving 0.016 mL of bevacizumab intravitreal injection using conventional needle. The HA group receiving 0.016 mL of bevacizumab intravitreal injection using HA-coated needle.

^a^Student’s *t*-test.

### Aqueous and vitreous humor levels of cytokines

The cytokine levels measured in the aqueous and vitreous humor after 12 intravitreal injections over 1 week are shown in Table 2. There were no significant differences in the aqueous and vitreous prostaglandin E_2_ (PGE_2_) levels among the three groups. There were no significant differences in the aqueous and vitreous interferon (INF)-γ levels among the three groups. Moreover, there were no significant differences in the aqueous and vitreous tumor necrosis factor (TNF)-α levels among the three groups. For aqueous interleukin (IL)-1β, IL-4, IL-6, and IL-17 levels, there were no significant differences among the three groups. Similarly, for the vitreous IL-1β, IL-4, IL-6, and IL-17, there were also no significant differences among the three groups. Conversely, the expression levels of aqueous IL-8 were significantly increased in the control group (850.9 ± 37.7 pg/mL) compared to the normal (525.2 ± 40.6 pg/mL) and HA needle (558.59 ± 17.5 pg/mL) groups, and vitreous IL-8 expression also significantly increased in the control group (479.1 ± 5.6 pg/mL) compared to the normal (373.4 ± 63.0 pg/mL) and HA needle (340.6 ± 13.3 pg/mL) groups.

**Table 2:** Comparison of aqueous and vitreous humor levels of cytokines among normal, control, and hyaluronic acid (HA) needle groups.

	Normal	Control	HA needle	P value^a^
**Aqueous humor level of cytokines, pg/mL**				
PGE_2_	63.4 ± 18.8	102.7 ± 23.2	98.4 ± 51.9	0.263
INF-γ	14.2 ± 1.2	15.2 ± 1.2	15.4 ± 1.3	0.346
TNF-α	44.1 ± 4.5	34.5 ± 4.8	40.8 ± 13.2	0.318
IL-1β	34.5 ± 11.8	36.5 ± 7.7	39.2 ± 10.5	0.807
IL-4	29.1 ± 7.0	31.9 ± 3.2	26.4 ± 15.1	0.734
IL-6	1.8 ± 0.5	1.8 ± 0.1	1.8 ± 0.3	0.985
IL-8	525.2 ± 40.6	850.9 ± 37.7	558.5 ± 17.5	< 0.001
IL-17	8.6 ± 1.6	6.9 ± 3.3	7.8 ± 2.7	0.643
**Vitreous humor level of cytokines, pg/mL**				
PGE_2_	54.1 ± 4.7	66.2 ± 11.4	77.0 ± 22.0	0.141
INF-γ	8.2 ± 1.6	5.2 ± 3.0	4.4 ± 4.1	0.244
TNF-α	50.8 ± 8.4	63.3 ± 8.8	59.1 ± 4.4	0.104
IL-1β	78.0 ± 11.8	56.4 ± 15.8	65.6 ± 15.1	0.156
IL-4	31.8 ± 6.5	27.4 ± 13.7	21.0 ± 11.9	0.420
IL-6	2.0 ± 0.5	1.7 ± 0.1	1.5 ± 0.1	0.097
IL-8	373.4 ± 63.0	479.1 ± 5.6	340.6 ± 13.3	0.001
IL-17	12.4 ± 2.6	11.5 ± 1.4	11.5 ± 5.2	0.920

Data are expressed as the mean ± standard deviation (SD) of 4 rabbits.

The HA group receiving 12 intravitreal injections using HA-coated needle over seven days. The control group receiving 12 intravitreal injection using conventional needle over seven days. The normal group no receiving intravitreal injection.

*PGE*_*2*_ prostaglandin E_2_, *INF* interferon, *TNF* tumor necrosis factor, *IL* interleukin.

^a^One-way analysis of variance with post-hoc Tukey’s honestly significant difference test.

## Discussion

This study demonstrated that intravitreal injections performed with HA-coated needles resulted in less extraocular regurgitation than conventional needles. The causes of intraocular infection after intravitreal injection include needle contamination by bacteria or conjunctival infection around the injection site. Previous studies analyzing causative organisms of intraocular infection found that most infections are caused by *Staphylococcus aureus* residing on the ocular surface introduced during intravitreal injection or via the passage remaining after injection^21,22^. Other studies reported that one-third of patients treated with intravitreal injections of 0.05 mL anti-VEGF showed temporary fluid-filled subconjunctival blebs during needle removal^23^. These blebs were caused by regurgitation of liquefied vitreous humor or injected drugs^24^. Vitreous regurgitation and incarceration induced at the injection site are risk factors for endophthalmitis after intravitreal injection^10^. In the present study, we found that when conventional needles were removed after intravitreal injections of ICG dye, either the dye leaked out of the eyeball or subconjunctival blebs formed in more than half of eyes. In contrast, the HA-coated needles developed for the present study prevented drug regurgitation by immediately closing the hole after removing the needles. Based on these results, the use of self-sealing intravitreal injection needles could minimize complications after injections, such as reflux of vitreous humor and drugs.

In the present study, anti-VEGF levels significantly increased in the aqueous humor one day after injection and in the vitreous humor one and three days after injection in the HA needle group compared to the control group. In a prior animal study, the half-life of bevacizumab in rabbit vitreous cavities was 4.32 days^25^. Similarly, in the present study we observed a decreasing tendency in anti-VEGF levels over time. Thus, it is considered reasonable that no significant differences were observed in levels at seven days after injection. These results suggest that HA-coated needles immediately closed the fistulae produced in eyeballs by intravitreal injection, which maintained higher vitreous anti-VEGF levels.

Although HA is safely used for various intraocular surgeries, such as cataract surgery, there may be safety concerns when HA-coated syringe needles are used for intravitreal injections. In this study, we observed no differences in the expression levels of eight cytokines measured in the aqueous and vitreous humor after 12 repeated intravitreal injections with HA-coated needles over a period of one week in comparison with a non-injected group. In contrast, aqueous and vitreous IL-8 levels markedly increased after repeated injections with conventional needles compared to normal and HA needle groups. IL-8 is a chemokine produced in macrophages and known to be involved in endophthalmitis progression ^26^. Deshmukh et al.^27^ reported that the pathogenesis of endophthalmitis is associated with increases in vitreous IL-1RA, IL-6, IL-8, GRO, and G-CSF levels. Thus, our results indicate that repeated intravitreal injection with conventional needles increases the risk of ocular inflammation, such as endophthalmitis, but HA-coated needles may prevent its occurrence.

When the HA-coated needle was inserted and then removed, HA remained at the injection site and blocked the hole in most cases. The mechanism of action is solid-to-gel phase transition of the coated HA. In general, the volume changes when a solid HA film is hydrated more than 10 times. Thus, the large volume changes is the most important factor in leakage prevention. However, we did not evaluate whether the HA actually enters the vitreous cavity, and whether floaters could be caused or whether HA would dissolve. HA is a major constituent of the vitreous humor, is biodegradable, and has been used for ocular drug delivery^28^. A previous study demonstrated that when a gel system incorporating HA was injected into the vitreous cavity, sustained release of the drug occurred^29^. Therefore, HA could potentially cause floaters if it enters the eye when intravitreal injections are performed with HA-coated needles, although HA is degraded in the eye.

The conventional methods used to prevent regurgitation of vitreous humor and injected drugs after intravitreal injection and reduce the risk of inflammation include pressing the injection site with a cotton swab immediately after removing the needle, using a thinner gauge needle, using a beveled scleral incision technique, and lowering IOP before injection^2,30^. Applying pressure to the injection site with a cotton swab after removing the needle is the most common method of reducing regurgitation of vitreous humor^10^. However, in vitreous incarcerations caused by a fistula formed during removal of the needle from the eyeball, this pressing method cannot eliminate the incarceration, and may cause infection after intravitreal injection^10^. Previous studies reported that if vitreous incarceration occurs in patients with uncomplicated intracapsular cataract extraction, severe inflammation can occur two weeks or more after surgery. Moreover, pressing with a swab carries risk of cotton fibers entering the vitreous cavity^31,32^.

Prior studies have shown that regurgitation of vitreous humor after intravitreal injection can be reduced using low-gauge needles^24,33^. In the present study we used 30-gauge needles in both the HA needle and control groups and found that drug regurgitation was significantly reduced in the HA needle group despite the increase in needle diameter caused by coating conventional needles with HA. Even if a needle thinner than 30 gauge is used, the HA-coated needle is expected to prevent extraocular regurgitation of vitreous humor and injected drug by closing the hole generated during needle removal.

All intravitreal injections in this study penetrated the sclera vertically (conventional straight incisions) to evaluate the effect of the HA-coated needle itself on blockage of vitreous reflux, as oblique or beveled scleral incision techniques cause less vitreous reflux compared to conventional straight incisions^2,30^. Although more vitreous reflux occurs after conventional intravitreal injection, straight incisions have the advantage of being easier and more convenient than oblique or beveled incision techniques. Therefore, a straight incision using the HA-coated needle can be performed easily and conveniently while preventing vitreous reflux. However, in this study, we did not compare vitreous reflux between the beveled and straight incision techniques with a HA-coated needle, so we could not investigate the superiority of different methods.

The cause of vitreous regurgitation and incarceration at the intravitreal injection site is believed to be an increase in IOP caused by the injected drug’s mass effect^10^. Hoang et al.^34^ conducted an animal study and showed that the regurgitation of injected drug could be reduced in rabbits by lowering IOP before injection^24^. However, there are limitations to lowering IOP before injection. Although it is possible to reduce IOP via paracentesis before intravitreal injection, applying a needle to the pars plana when IOP is low is risky because it is difficult for the needle to penetrate the sclera^10^, and paracentesis itself may be another risk factor for infection^35^.

This study has some limitations. First, this study did not measure IOP following intravitreal injection. The increase in IOP immediately after intravitreal injection is proportional to the amount of drug injected into the eye and inversely proportional to vitreous reflux^36^. Therefore, if IOP was measured immediately after intravitreal injection, IOP would have been higher in the HA group than in the control group. However, the IOP increase caused by intravitreal injection leads to a transient decrease in ocular perfusion pressure that does not impair retinal blood flow^37^. Second, this study did not perform a histological study to investigate how long HA remains in the hole created by intravitreal injection. Considering the half-life of HA, it will be necessary to further explore this topic.

## Conclusion

This study demonstrated that HA-coated needles can close ocular fistulae. Using HA-coated needles can prevent vitreous incarceration and extraocular drug regurgitation after intravitreal injections by immediately closing the hole formed after needle removal. Our findings imply that the use of HA-coated needles can prevent outflow of vitreous humor and drugs through the needle passage site.

## Methods

Ninety healthy New Zealand white rabbits weighing 2.0–2.2 kg were randomly divided into 3 groups (HA needle, control, and normal groups)^38^. Rabbits in the HA group (n = 40) were injected using 30-gauge HA-coated self-sealing intravitreal injection needles, while rabbits in the control group (n = 40) were injected using 30-gauge conventional needles. All intravitreal injections were conducted using a conventional straight incision. The 10 untreated rabbits were allocated into the normal group. For general anesthesia, 5 mg/kg body weight (BW) xylazine hydrochloride (Rompun 2%, Bayer, Leverkusen, Germany) was injected intramuscularly and 5 mg/kg Alfaxalone (Alfaxan; Jurox Pty Ltd, Rutherford, NSW, Australia) was administered intravenously. Repeated application of 2–3 drops of 0.5% proparacaine hydrochloride (Alcaine; Alcon Laboratories, Fort Worth, TX) was performed for topical anesthesia. After the experiment, experimental rabbits were humanely euthanized using a CO_2_ chamber with a gradual-fill method under general anesthesia^38^. This study was conducted in accordance with the Statement for the Use of Animals in Ophthalmic and Vision Research (ARVO) and ARRIVE guideline^39,40^. The study protocol was approved by the Institutional Animal Care and Use Committee of Korea University College of Medicine, Seoul, Republic of Korea.

### Preparation of self-sealing intravitreal injection needles

Two important criteria to achieve self-sealing needles for intravitreal injection are ‘leakage prevention efficacy’ and ‘low friction during insertion.’ Collagen, alginate, gelatin, carboxymethyl cellulose, dextran, and HA were tested. Subsequently, we performed studies to find the relationship between molecular weight of HA and self-sealing efficacy. We chose 10-, 200-, 700-, and 1,000-kDa HAs.

For the experiment, 15 mg of HA, with a molecular weight of 700 kDa was dissolved in 1 mL of distilled water to prepare self-sealing intravitreal injection needles. Oxygen plasma treatment was applied to 30-gauge needles (BD PrecisionGlide™ Needles) of the type commonly used for intravitreal injection for 10 min, and then the needles were coated with 4.6 μL of HA solution at room temperature for an hour while rotating. This process was repeated twice to prepare the self-sealing injection needles. The needle’s front ends were coated thinly, while the back ends were coated thickly to improve their scleral penetration and the HA coating’s sealing effect. The HA-coated self-sealing intravetreal injection needles was analyzed using Field Emission Scanning Electron Microscope (FE-SEM) (JSM-IT800, JEOL, Japan). In addition, atomic species of the HA-coated and HA-uncoated layer on the needle surface were analyzed with energy-dispersive spectroscopy (EDS) in tandem with SEM.

### Penetration test of HA-coated self-sealing intravitreal injection needles

A medical needle puncture force tester (CL15811-E; Shanghai Yuanzi Electronic Technology Co., Ltd, Shanghai, China) was used to investigate the penetration force of the HA-coated self-sealing injection needles (30 gauge) on living tissue. This experiment was conducted independently three times.

### Immediate regurgitation of dye after intravitreal injection

**Figure 6:**
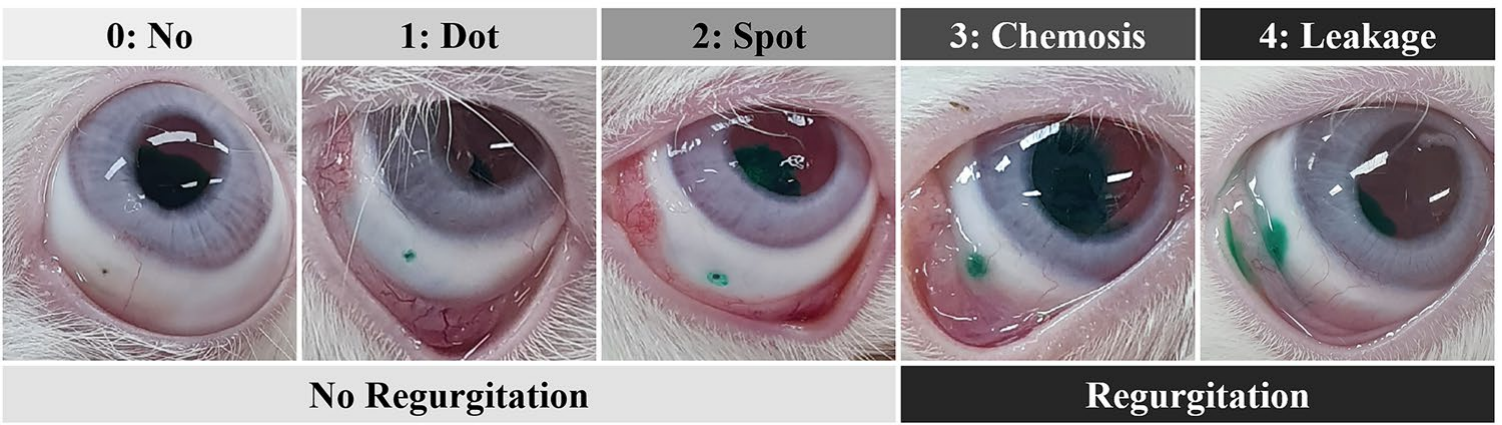
Standard photos used for evaluating the degree of dye leakage after intravitreal injection. Staining status in the injection site’s conjunctiva was rated on a scale of 0 to 4 (0, no stain; 1, dot stain; 2, spot stain; 3, chemosis; 4, leakage). A rating of 0–2 was defined as having no dye leakage, while a rating of 3–4 was defined as having dye leakage.

To evaluate the immediate regurgitation of injected drugs after intravitreal injection, we investigated the degree of ICG dye leakage around the injection site within 10 s after ICG dye (0.050 mL) injection into the vitreous cavities of rabbits using HA needles (*n* = 18) and controls (*n* = 18). All injections were performed using a conventional straight incision under general anesthesia by a single investigator (J.Y.H.). Staining or leakage status in the injection site’s conjunctiva was rated on a scale of 0 to 4 (0, no stain; 1, dot stain; 2, spot stain; 3, chemosis; 4, leakage) by another blinded investigator (Y.E.). A rating of 0–2 was defined as having no dye leakage, while a rating of 3–4 was defined as having dye leakage (Fig. 6).

### Aqueous and vitreous humor levels of anti-VEGF

A micropipette was used to accurately obtain the amount of anti-VEGF injected intraocularly^41^. The levels of anti-VEGF in the aqueous humor and vitreous humor were measured after the injection of 0.016 mL of bevacizumab (Avastin, Roche Pharma) into the rabbit’s vitreous cavity 2 mm from the limbus of each eye using HA-coated (HA needle group; *n* = 18) and conventional (control group; *n* = 18) needles with a 1-ml syringe. The concentrations of aqueous and vitreous anti-VEFG were measured one, three, and seven days after injection (n = 6 at each time point for each group), and were also determined in eyes in which intravitreal injection was not performed (normal group; *n* = 6). For this measurement, the animals were euthanized one, three, and seven days after intravitreal injection, and their eyeballs were then extracted and frozen. The aqueous humor and vitreous humor were separated from the eyeballs while frozen, before thawing and homogenization^42^. While the thawed aqueous humor (50 or 100 µl) was used for enzyme-linked immunosorbent assay (ELISA), the vitreous humor was centrifuged at 1,000 rpm for 20 min, and the resulting supernatant (50 or 100 µl) was used for analysis. Anti-VEGF levels in the aqueous and vitreous samples were measured using modified ELISA^43,44^. A 96-well plate was coated with 100 µl/mL recombinant human VEGF165 (R&D Systems) overnight at 4 °C and washed three times with phosphate-buffered saline (PBS) containing 0.05% Tween-20. Then, 3% bovine serum albumin (BSA)/PBS was applied overnight at 4 °C (200 µL/well) to block the well. After washing five times with PBS containing 0.05% Tween-20, aqueous or vitreous samples diluted in 0.1% BSA/PBS was added to the well plates overnight at 4 °C (50 µL/well). Then, 1 µg/mL horseradish peroxidase (HRP)–goat anti-human IgG (H + L) conjugate (Invitrogen Corporation, Carlsbad, CA) was applied for a 3-h incubation period at room temperature. After washing five times, 100 µL 3,3',5,5'-tetramethyl benzidine (TMB) substrate was applied and then 1 M hydrogen chloride (100 µL) was added to stop the reaction. The optical density was measured at 450 nm using a microplate spectrophotometer (Spectramax Plus 384; Molecular Devices, Sunnyvale, CA, USA).

### Aqueous and vitreous humor levels of cytokines

**Figure 7:**
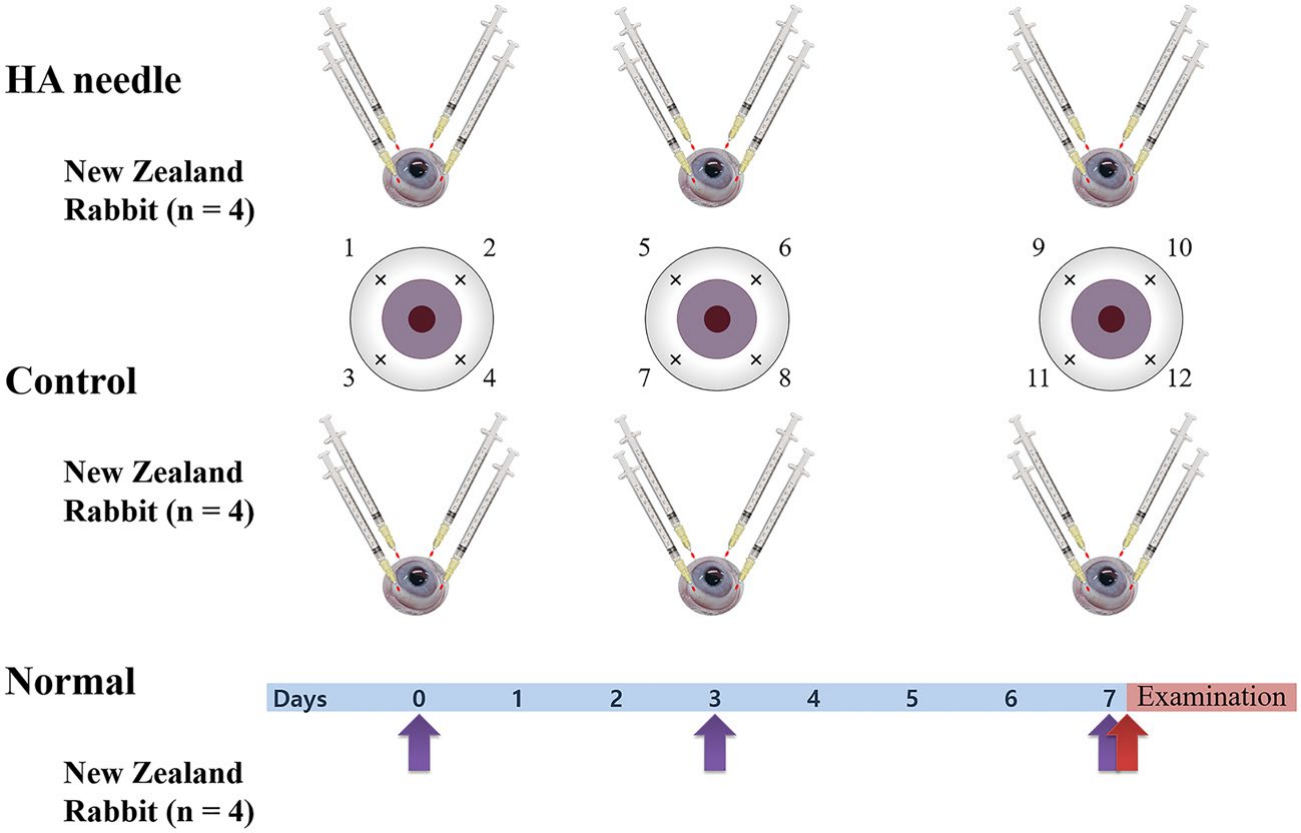
The experimental protocol for comparing aqueous and vitreous humor levels of cytokines after 12 intravitreal injections among HA needle, control, and normal groups. Experimental groups are presented in Table 2.

In this study, four intravitreal injections (superotemporal, superonasal, inferotemporal, and inferonasal areas) were performed 2 mm from the limbus of each rabbit eye using HA-coated (HA needle group; *n* = 4) and conventional (control group; *n* = 4) needles to investigate whether repetitive intravitreal injections caused inflammation. The sclera was pierced and nothing injected repetitively in the same way three and seven days after the initial injection (Fig. 7). Rabbits treated with 12 intravitreal injections were then euthanized on the day of the last injection and their eyeballs extracted. As described for the previous experiment, the eyeballs were frozen before separating the aqueous and vitreous humor to measure expression levels of inflammatory cytokines. Inflammatory cytokine levels were measured even in eyes (normal group; *n* = 4) that did not receive intravitreal injections. While thawed aqueous humor (50 or 100 µl) was used for ELISA, the vitreous humor was centrifuged at 3,000 rpm for 20 min, and the resulting supernatant (50 or 100 µl) was used for analysis. This cytokine measurement was conducted according to the manufacturers’ protocols using commercial ELISA kits: PGE_2_ (MBS763445, MyBioSource), INF-γ (MBS2510723, MyBioSource), TNF-α (DY5670, R&D Systems), IL-1β (MBS2702039, MyBioSource), IL-4 (MBS763238, MyBioSource), IL-6 (MBS731230, MyBioSource), IL-17 (MBS7606866, MyBioSource), and IL-8 (MBS, MyBioSource).

### Statistical analyses

All data were analyzed with the Statistical Package for Social Sciences Statistics Standard 20 (IBM Corp., Armonk, NY, USA). Chi-square tests were used to assess differences in immediate regurgitation of ICG dye between the control and HA needle groups after intravitreal injections. Student’s *t*-test was used to compare aqueous and vitreous humor levels of anti-VEGF between the control and HA needle groups. One-way analysis of variance (ANOVA) with post-hoc Tukey’s honestly significant difference (HSD) test was used to compare aqueous and vitreous levels of cytokines between normal, control, and HA needle groups. A *p*-value < 0.05 was considered statistically significant.
